# Weighted Bayesian Poisson Regression for The Number of Children Ever Born per Woman in Bangladesh

**DOI:** 10.1007/s44199-022-00044-2

**Published:** 2022-06-14

**Authors:** Jabed H. Tomal, Jahidur Rahman Khan, Abdus S. Wahed

**Affiliations:** 1 Department of Mathematics and Statistics, Thompson Rivers University, Kamloops, British Columbia Canada; 2 Discipline of Paediatrics, School of Clinical Medicine, University of New South Wales, Sydney, Australia; 3 Department of Biostatistics, Graduate School of Public Health, University of Pittsburgh, Pennsylvania, USA

**Keywords:** Poisson regression, Bayesian method, Weighted likelihood, Fertility rate, Bangladesh

## Abstract

Number of children ever born to women of reproductive age forms a core component of fertility and is vital to the population dynamics in any country. Using Bangladesh Multiple Indicator Cluster Survey 2019 data, we fitted a novel weighted Bayesian Poisson regression model to identify multi-level individual, household, regional and societal factors of the number of children ever born among married women of reproductive age in Bangladesh. We explored the robustness of our results using multiple prior distributions, and presented the Metropolis algorithm for posterior realizations. The method is compared with regular Bayesian Poisson regression model using a Weighted Bayesian Information Criterion. Factors identified emphasize the need to revisit and strengthen the existing fertility-reduction programs and policies in Bangladesh.

**Supplementary Information:**

The online version contains supplementary material available at 10.1007/s44199-022-00044-2.

## Introduction

During the last century, the world has experienced an exponential growth of its human population increasing from 1.65 billion in 1900 to 6 billion in 1999 [14, 71]. Even though the current (2021) world population is approximately 7.88 billion, it is unlikely that the world population growth is going to be stabilized in this century [33]. With such a huge population growth of one species over a long period of time risks the planet to become economic, environmental, and socially unsustainable [12]. On the other hand, there is substantial variation in human population growth by country. When some of the countries in the developed world are experiencing a steady decline in their population growth, the decline in the developing countries are yet to be satisfactory [20].

Population dynamics in a country is an important tool for the government and non-government organizations for planning and policy making. Among many forces that have been driving population dynamics, the most important determinant is the fertility [71]. Fertility rates in countries around the globe have gone down over the last decades. Globally, the total fertility rate (TFR) has declined from 3.2 (average number of live birth per woman during reproductive age) in 1990 to 2.5 in 2019 [97]. However, there is a substantial variation in the decline of fertility rates across the countries. For example, the countries in Sub-Saharan Africa have experienced a decline in fertility from 6.3 in 1990 to 4.6 in 2019. During the same period of time, fertility rates have declined from 4.4 to 2.9 in Northern Africa and Western Asia, from 4.3 to 2.4 in Central and Southern Asia, from 2.5 to 1.8 in Eastern and South-Eastern Asia, from 3.3 to 2.0 in Latin America and the Caribbean, and from 4.5 to 3.4 in Oceania (excluding Australia and New Zealand). The fertility rate was already below 2.0 in 1990 in Australia, New Zealand, Europe, and North America which has remained steady up to 2019 [97].

Bangladesh, a country in South Asia, has experienced a steady decline in its fertility rate over years: specifically, a reduction from 4.5 in 1990 to 2.3 in 2019 [38]. Even though Bangladesh has undergone a sharp decline in its fertility rate over time, the rate has become relatively stable in recent years [42]. Given the fact that Bangladesh is the eighth largest country in the world in terms of both population density and size, residing in its relatively small landmass, there is still a need for further reduction of the rate to achieve the 4th Health, Population and Nutrition Sector Program (HPNSP) target^1^ of two children per woman by 2022: The health promotion and risk reduction target set forth by the World Health Organization and Government of Bangladesh. In addition to other measures of fertility such as TFR and general fertility rate (GFR) [7], the number of children ever born (CEB) to a woman forms a core component of fertility and, thus, population dynamics in any country [78, 87], as it provides a complete picture of fertility behaviour by taking into consideration the lifetime fertility of women.

The focus of this paper is to use novel survey-weighted Bayesian Poisson regression model to understand the roles of individual, household, community, and regional factors that explain CEB which is important for devising policies to further reduce fertility in Bangladesh. Previous studies linked CEB to woman’s age, age at marriage, education, husband’s education and religion [1, 61]. Infant/child death, woman’s age, religion, husband’s education and the practice of family planning were reported to have positive effects on fertility, where woman’s education and employment status had negative effects [76]. Additionally, Kiser and Hossain [61] identified religion, wealth index, whether the last child was wanted or not, and division as other significant factors of CEB. Nahar and Zahangir [79], also using 2014 BDHS data, found that the fertility rate among ever-married women of reproductive age was higher among women who were Muslim, illiterate, or primary school graduate; had no access to mass media; gave first birth at the age of 15 years or earlier; ever used any contraceptive; and desired three or more children. In their study, place of residence, division, husband’s education, woman’s working status, husband’s occupation, age at first marriage, and spousal age difference were found as the other important factors of CEB. Although several studies in Bangladesh have assessed the relationship between individual, household, community level factors to explain CEB (and fertility), there is limited research, if any, in Bangladesh to assess the relationship between social factors and fertility which are found to be stronger predictors of fertility behaviour in other countries [5]. For example, in Amara [5], regional poverty rate was positively, while regional unemployment rate and the availability of health centers were negatively related to CEB. In short, it is important to further assess the influence of individual, household, regional, and societal factors on fertility behaviour of women in Bangladesh.

Poisson distribution is a popular choice to model a count variable where its mean and variance are represented by the rate of occurrence of an event [53]. The event of interest, in this study, is the birth of a child by a married woman of reproductive age in Bangladesh. Poisson regression allows modeling of relationship between a count variable of interest (i.e., response) and explanatory variables via a log-linear link function where the log of the conditional mean of the response is expressed as a function of the explanatory variables [16]. El-Sayyad [27] reviewed a classical Poisson regression model where the response variable of interest was considered to follow a Poisson distribution. Frome [30] showed how to estimate the rate of occurrence of an event using a Poisson regression model and proposed iteratively reweighted least squares method to estimate the parameters. Lawless [65] examined the Negative Binomial and Mixed Poisson Regression models to deal with extra-Poisson variation in regression analysis for count data. Consul and Famoye [22] proposed a generalized Poisson distribution to show its usefulness in fitting over-dispersed and under-dispersed count data. Lambert [63] proposed a zero-inflated Poisson regression model for count data with excess of zeros. Newton and Raftery [83] introduced weighted likelihood bootstrap to simulate from a weighted distribution by calculating the maximum likelihood estimator using method such as iteratively reweighted least squares. Wang [99] proposed a maximum weighted likelihood method to combine data from different sources, and proved consistency and asymptotic normality properties of the estimators. Following the ideas presented in [83] and [99], we have proposed a weighted likelihood function for our fully parameterized Poisson regression model.

In contrast, Bayesian Poisson regression combines information from the data and knowledge extracted from experts’ beliefs via likelihood function, prior, and posterior distribution of the model parameters [32]. El-Sayyad [27] proposed a Bayesian Poisson regression model and compared its performance with the classical Poisson regression model. Tsionas [96] proposed a regression model for the multivariate Poisson distribution, and employed Bayesian methods for inference based on Gibbs sampling and data augmentation. Christiansen and Morris [19] proposed a hierarchical Poisson regression model, and Kim et al. [59] developed the corresponding Bayesian hierarchical Poisson regression model and showed applications to a driving study with kinematic events to understand the driving behavior of teenagers in the early months after licensure. While there are studies on geographically weighted Poisson regression models [29, 44, 81, 90], classical likelihood-based weighted generalized linear model [83], and cluster-weighted generalized linear model [49], there is no literature on likelihood-based fully Bayesian weighted Poisson regression models.

In this paper, we propose a likelihood-based Bayesian Poisson regression model when the likelihood contribution from an observation is adjusted by its weight to model the number of CEB to married women of reproductive age in Bangladesh. We illustrated our proposed model with several prior distributions [9] and showed how these priors can be used to shrink model parameters (i.e., regularization; [95]) and thus to check robustness of the findings [24]. After deriving the posterior distribution for the model parameters, we showed how to to generate Markov chain Monte Carlo (MCMC) samples [34] using the Metropolis algorithm [45, 73]. We proposed a weighted Bayesian information criterion (WBIC) to be used for model selection [17]. The proposed model is used in analysing the Bangladesh Multiple Indicator Cluster Survey (MICS 2019) dataset to explain the CEB among married women of reproductive age in Bangladesh using various individual, household, administrative division, societal, and district level factors. While past such studies were based on data from smaller surveys, our study is based on the most recent, nationally representative large-scaled Bangladesh MICS 2019 data. In addition, this study also investigated the influence of societal factors which has never been explored, to the best of our knowledge, in earlier studies. The findings of our study will help to strengthen key family planning strategies and raise awareness among different stakeholders to achieve the goal set forth by HPNSP [37].

Thus, the major objectives of this study are to 1) use a novel weighted Bayesian Poisson regression model that incorporates survey weights to explain CEB in order to assess the effects of individual, household, regional factors on CEB, 2) describe the related inference and implementation process, and assess the robustness of the results across various priors, and 3) establish and assess the societal determinants of CEB using a recent, nationally representative, and large survey data from Bangladesh.

Section 2 describes the MICS 2019 data, factor variables, and sampling weights. Section 3 describes the methodology of Bayesian weighted Poisson regression method by introducing the model, the likelihood, prior, posterior, and the Metropolis algorithm. Subsection 3.2 discusses different types of priors and presents in details the Normal, Laplace and Cauchy priors. Section 3.5 presents the weighted Bayesian information criteria (WBIC). Computational details are provided in Sect. 4. Results from MICS 2019 data analysis is presented in Sect. 5. Section 6 presents the conclusion and discussion.

## MICS 2019 Data

Individual, household, regional data are obtained from the Bangladesh Multiple Indicator Cluster Survey of 2019 (MICS 2019) conducted by the joint efforts of the Bangladesh Bureau of Statistics and United Nations Children’s Fund [38]. In addition, societal data relating to district level literacy and contraceptive prevalence rates are extracted from the Bangladesh Sample Vital Registration System of 2018 (SVRS 2018) report [10]. We note that district (the 64 districts of Bangladesh are depicted in Fig. F3a) is the second administrative level unit in Bangladesh. In terms of landmass, the largest and the smallest districts are Rangamati and Narayanganj with sizes 6116 and $700 \; \text {km}^2$, respectively. Moreover, according to the 2011 census, their estimated population sizes were about 596 and 2897 thousands, respectively. The Bangladesh MICS 2019 is a cross sectional and nationally representative survey which has used a two-stage stratified cluster sampling method to draw the sample. Each of the survey respondents is assigned a sampling weight, to be adjusted in the analysis, in order to calculate population level estimates. The weight for individual woman is the household weight (inverse of its selection probability multiplied by the inverse of the household response rate) multiplied by the inverse of the individual response rate in the stratum. That is, larger selection probability (or response rate) imposes smaller weight and vice versa. Details of the survey and the sampling weights are available in the MICS 2019 report [38].

This MICS 2019 data contain information for a total of 68,709 women of reproductive age of $15-49$ years. An analytic sample of 51,361 women is selected after excluding women who were not married or never-married (13,940), women with missing values in the frequency of reading newspaper, listening to ratio, and watching television (3,364), and husband’s age (44). District level data from SVRS 2018 are spatially added to the individual level data set.

**Fig. 1:**
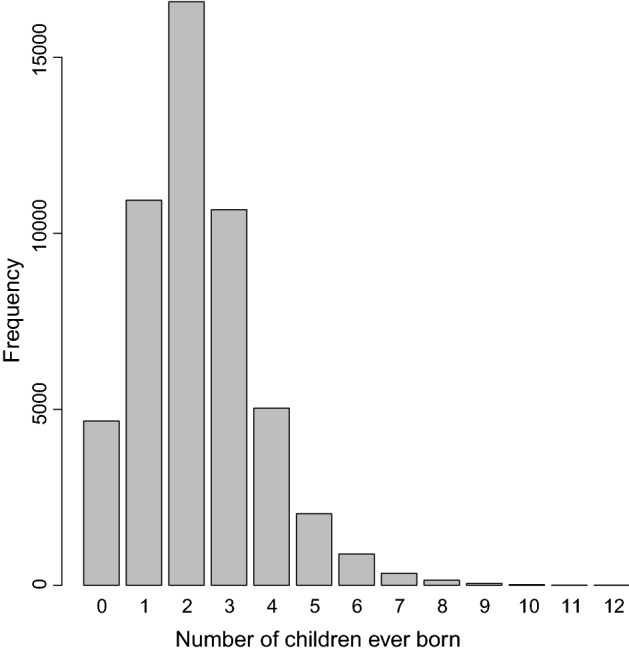
Bar-plot showing the distribution of the number of children ever born to married women of reproductive age in Bangladesh

In this study, the response variable is the number of children ever born (CEB) among currently married women of reproductive age in Bangladesh. Figure 1 shows the distribution of CEB which closely resembles a Poisson probability distribution [54]. The explanatory variables included in this study are woman’s age at marriage (in years), woman’s current age (in years, during interview), husband’s age (in years, during interview), woman’s education (preschool or none, elementary, high school, college or higher), woman’s exposure to media (yes, no), ethnicity (Bengali, others), household wealth index (poorest, poorer, middle class, richer, richest), area (rural, urban), district level adult literacy rate ($\%$) and contraceptive prevalence rate ($\%$), and administrative division (Barishal, Chittagong, Dhaka, Khulna, Mymensingh, Rajshahi, Rangpur, Sylhet). The visuals of the 8 divisions can be achieved from Fig. F3b. Woman’s exposure to mass media is assessed based on whether she has exposure to any of the three activities such as listening to the radio, watching television, and reading newspaper (yes); otherwise (no). The categories for the household wealth index variable are calculated based on quintiles of the principal component analysis of various asset variables [38]. Adult literacy rate is defined as the percentage of population of age 15 years-and-over who can write letters to their peers, and contraceptive prevalence rate is defined as the percentage of couples currently practicing (during interview) any contraceptive methods to the number of currently married women of reproductive age [10].

## Bayesian Weighted Poisson Regression

### The Model and the Likelihood Function

Let *Y* be the number of children ever born (CEB) to a married woman of reproductive age in Bangladesh. As in [54], we assume that the conditional distribution of *Y* given the observed vector of explanatory variables $\mathbf {x}$ follows a Poisson distribution

1 $$\begin{aligned} \begin{array}{ccc} P(Y = y| \mathbf {x}, \lambda (\mathbf {x}; \; \varvec{\beta })) = \frac{e^{-\lambda (\mathbf {x}; \; \varvec{\beta })}\lambda (\mathbf {x}; \; \varvec{\beta })^y}{y!}&\text {for}&y = 0, 1, 2, 3, \ldots , \end{array} \end{aligned}$$

where $\lambda (\mathbf {x}; \; \varvec{\beta })$ is the conditional mean and variance of *Y* given $\mathbf {x}$. As is customary in Poisson regression [16], we use a log-link to connect the linear predictor to the mean function. More specifically,

$$\begin{aligned} \ln \left[ E(Y|\mathbf {x}, \varvec{\beta })\right] = \ln (\lambda (\mathbf {x}; \; \varvec{\beta })) = \mathbf {x}^T \varvec{\beta }, \end{aligned}$$

where $\varvec{\beta } = \left( \beta _0, \beta _1, \beta _2, \ldots , \beta _p\right) ^T$ and $\mathbf {x} = \left( 1, x_1, x_2, \ldots , x_p\right) ^T$ are $(p+1)$ column vectors of regression coefficients and explanatory variables, respectively.

Let $w_i$ be the weight for the *i*th woman with observed response $y_i$ and vector of explanatory variable values $\mathbf {x}_i$. Then, following the idea presented in [83] and [99], we define the weighted likelihood function as

2 $$\begin{aligned} L(\varvec{\beta }) = \prod _{i=1}^n P\left( Y_i = y_i| \mathbf {x}, \lambda (\mathbf {x}; \; \varvec{\beta })\right) ^{w_i}, \end{aligned}$$

where $P\left( .\right)$ is the probability mass function defined in Eq. 1.

### Prior

We denote the prior distribution, which reflects our prior belief, for $\varvec{\beta }$ as $\pi (\varvec{\beta })$. There are many types of priors available in Bayesian literature [32]. The vague, flat or diffuse prior is a non-informative prior [66] which is used to reflect no-prior knowledge about the parameter of interest. Another type of non-informative and scaled-invariant prior is Jeffrey’s prior [52, 91], often applied to a single parameter, which is obtained as $\pi (\varvec{\beta }) \propto \sqrt{\left| I(\varvec{\beta })\right| }$, where $I(\varvec{\beta })$ is the Fisher information matrix. The unit information prior is a weakly informative prior which contains information of a single data point [47]. In addition, the spike-and-slab prior [77] is useful for variable selection particularly when the number of explanatory variables is larger than the number of data points. On the other hand, an informative prior reflects our current knowledge and uncertainly of the parameter of interest [9]. In this paper, we use carefully designed, well justified and communicated three informative priors.

Assuming independence among the regression coefficients $\beta _j \ (j = 0, 1, \ldots , p)$ [11], the joint prior distribution for $\varvec{\beta }$ is formulated as

3 $$\begin{aligned} \pi (\varvec{\beta }) = \prod _{j = 0}^p \pi (\beta _j|\mu _j, \sigma _j), \end{aligned}$$

where $\pi (\beta _j|\mu _j, \sigma _j)$ is the marginal prior for the jth coefficient. For $\pi (\beta _j | \mu _j, \sigma _j)$, three different types of priors are considered.

First, we have considered a Normal prior [67] with density

$$\begin{aligned} \ \pi (\beta _j|\mu _j, \sigma _j) = \frac{1}{\sqrt{2\pi }\sigma _j} \exp \left\{ -\frac{1}{2}\left( \frac{\beta _j - \mu _j}{\sigma _j}\right) ^2\right\} , \end{aligned}$$

where the location and scale parameters are $\mu _j$ and $\sigma _j$, respectively. To impose a constraint similar to *L*2 norm that performs regularization like a Ridge regression [46], all $\mu _j$’s are set to be 0.

The second prior distribution we have used is a Laplace prior [102] with density

$$\begin{aligned} \ \pi (\beta _j|\mu _j, \sigma _j) = \frac{1}{2\sigma _j} \exp \left\{ -\frac{\left| \beta _j - \mu _j\right| }{\sigma _j}\right\} , \end{aligned}$$

where the location and scale parameters are $\mu _j$ and $\sigma _j$, respectively. This prior is also known as double exponential prior, which acts as *L*1 norm constraint when $\mu _j = 0$ and performs regularization like the LASSO [95].

The third prior we considered is a Cauchy distribution [35] with density

$$\begin{aligned} \ \pi (\beta _j|\mu _j, \sigma _j) = \left[ \pi \sigma _j \left( 1 + \left( \frac{\beta _j - \mu _j}{\sigma _j}\right) ^2\right) \right] ^{-1}, \end{aligned}$$

where the location and scale parameters are $\mu _j$ and $\sigma _j$, respectively. When $\mu _j = 0$, this prior imposes a greater shrinkage than the Normal and Laplace priors [86].

**Fig. 2:**
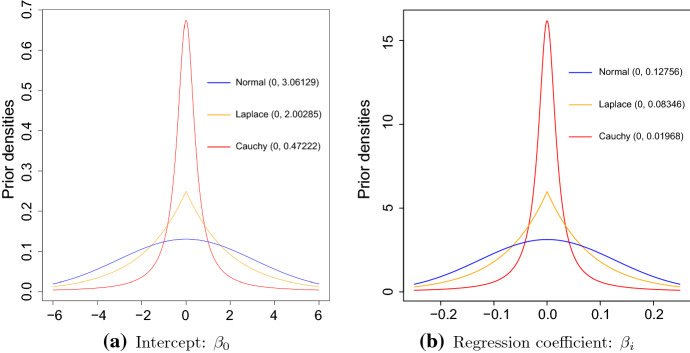
The Normal, Laplace and Cauchy priors. The left and right panels are for the intercept ($\beta _0$) and regression coefficient ($\beta _i$), respectively. The location parameter in each case is $\mu = 0$. The scale parameters are so chosen that the probability coverage for both the intercept and regression coefficient over the interval $(-6, +6)$ for $\beta _0$ and $(-0.25, +0.25)$ for $\beta _i$ is 0.95

Figure 2 visualizes the three priors. The flattest (blue) and the most peaked (red) curves are for the Normal and Cauchy priors, respectively, whereas the Laplace prior is in the middle (purple). It is clear that the Cauchy prior will apply the most shrinkage followed by the Laplace and Normal priors.

### Posterior

The joint posterior distribution of $\varvec{\beta }$ is obtained by combining the likelihood function (Eq. 2) and the prior distribution (Eq. 3) as follows:

4 $$\begin{aligned} \pi (\varvec{\beta }|\mathbf {y}, \mathbf {x}) \propto L(\varvec{\beta }) \times \pi (\varvec{\beta }) = \prod _{i=1}^n P\left( Y_i = y_i| \mathbf {x}_i, \lambda (\mathbf {x}; \; \varvec{\beta })\right) ^{w_i} \times \prod _{j = 0}^p \pi (\beta _j|\mu _j, \sigma _j), \end{aligned}$$

where *L*(.) and $\pi (.)$ are the likelihood function and the prior distribution, respectively. The joint posterior distribution of $\varvec{\beta }$ does not have a closed form expression of a standard statistical distribution. Therefore, it is neither possible to use Monte Carlo method [74] nor a Gibbs sampler [31] to generate samples from the posterior. Instead, we propose the following Metropolis algorithm [73].

### The Metropolis Algorithm

We approximate the posterior distribution of $\varvec{\beta }$ via the Metropolis algorithm [73]. For a given state of the parameter vector $\varvec{\beta }^{(s)}$, a new state is generated as follows:

1. Sample a proposal state $\varvec{\beta }^*$
  $$\begin{aligned} \ \varvec{\beta }^* \sim P^*(\varvec{\beta } | \varvec{\beta }^{(s)}), \end{aligned}$$
  where $P^*(.)$ is the proposal distribution to be explained later.
2. Calculate the acceptance ratio *r*
  $$\begin{aligned} \ r = \frac{\pi (\varvec{\beta }^*|\mathbf {y}, \mathbf {x})}{\pi (\varvec{\beta }^{(s)}|\mathbf {y}, \mathbf {x})}, \end{aligned}$$
  where $\pi (.|\mathbf {y}, \mathbf {x})$ is the posterior distribution of $\varvec{\beta }$.
3. Accept or reject the proposal with
  $$\begin{aligned} \ \varvec{\beta }^{(s+1)} = \left\{ \begin{array}{ll} \varvec{\beta }^* &{} \text {with probability} \ \min (r, 1)\\ \varvec{\beta }^{(s)} &{} \text {with probability} \ 1- \min (r, 1). \end{array}\right. \end{aligned}$$

The proposal distribution $P^*(.)$ for $\varvec{\beta }$ is considered as multivariate-normal (MVN):

$$\begin{aligned} \ \varvec{\beta } | \varvec{\beta }^{(s)} \sim \text {MVN}\left( \varvec{\beta }^{(s)}, \Sigma ^{p}\right) , \end{aligned}$$

where $\Sigma ^{p}$ is the proposal variance-covariance matrix as like the variance-covariance matrix of the ordinary least squared method

$$\begin{aligned} \ \Sigma ^{p} = \left[ \hat{\sigma }^2 \left( \mathbf {x}^T\mathbf {x}\right) ^{-1}\right] k, \end{aligned}$$

with $\hat{\sigma }^2$ be the sample variance of $\{\log (y_1 + 0.5), \log (y_2 + 0.5), \ldots , \log (y_n + 0.5)\}$. To determine an appropriate $\Sigma ^{p}$, we suggest to begin with $k = 1$. If this choice produces very low or high acceptance rate, we recommend to adjust $\Sigma ^{p}$ using other values of $k > 0$ until a reasonable acceptance rate is achieved.

### Weighted Bayesian Information Criterion

Akaike Information Criterion (AIC; [2]) and Bayesian Information Criterion (BIC; [92]) have been popular in statistical model selection for a long time [3, 82, 85]. In this study, aligned with the weighted likelihood function, we propose the following weighted Bayesian information criterion (WBIC) to compare the models,

$$\begin{aligned} \ \text {WBIC} = (p + 1) \ ln(n) - 2 \ ln(L(\hat{\varvec{\beta }})), \end{aligned}$$

where $p+1$, *n*, and $L(\varvec{\beta })$ denote the number of model parameters, data points, and the weighted likelihood function defined in Eq. 2, respectively. As always, the model with the lowest WBIC is preferred.

## Computational Details

First, we provide the computational details for the weighted Poisson regression model with the normal prior. The same procedure is followed by all other models and priors.

### Prior Specification

Inspired by the specification process of the null hypothesis in statistical literature [47], we considered the hyperparameter $\mu _j$ to be 0 for all $j = 0, 1, 2, \ldots , p$. Therefore, apriori, we considered no effect for any of the explanatory variables on CEB. In addition, such specification enables us to apply shrinkage to each coefficient towards 0 as detailed in Sect. 3.2.

Following the recommendation in [9], we used carefully chosen informative priors for our model. Noting that the maximum likelihood estimate of $\beta _0$ is $-2.547$, to ensure $95\%$ prior probability for $\beta _0 \in [-6, +6]$, we choose the scale hyperparameter $\sigma _0$ to be 3.06129, 2.00285, and 0.47222 for the Normal, Laplace and Cauchy priors, respectively. The elicitation of $\sigma _0$ is straightforward and its values are obtained using **R** [88] statements *qnorm*, *qlaplace* and *qchauchy* for the Normal, Laplace and Cauchy priors by providing the appropriate prior probability coverage. The values of $\sigma _0$ of 3.06129, 2.00285, and 0.47222 for the Normal, Laplace and Cauchy distributions ensure that the highest $95\%$ prior masses of $\beta _0$ are between $-6$ and $+6$. Similarly, noting that the maximum likelihood estimates for the regression coefficients $\beta _j \ (j = 1, \ldots , p)$ range from $-0.157$ and 0.226, to ensure a $95\%$ prior probability for each $\beta _j \in [-0.25, +0.25]$, we choose the scale hyperparameter $\sigma _j \ (j = 1, \ldots , p)$ to be 0.12756, 0.08346, and 0.01968 for the Normal, Laplace and Cauchy priors, respectively, for all $j = 1, \ldots p$. As like $\sigma _0$, the values of $\sigma _j$ are obtained using **R** [88] statements *qnorm*, *qlaplace* and *qchauchy* for the Normal, Laplace and Cauchy priors by providing the appropriate prior probability coverage. The values of $\sigma _j$ of 0.12756, 0.08346, and 0.01968 for the Normal, Laplace and Cauchy distributions ensure that the highest $95\%$ prior masses of $\beta _j$ are between $-0.25$ and $+0.25$. These prior distributions are depicted in Fig. 2. The Cauchy prior applies the most of the shrinkage towards 0 followed by Laplace and Normal priors. These priors will be used to check robustness [24, 25] of our results in Sect. 5.2.

### Burn-in, Thinning and Acceptance Rate

We determined the value of *k* using trial and error method. After few attempts, we optimized *k* to be 1/6 and ran the algorithm for a total of 105,000 times, which provided an acceptance rate [4] of $35.12\%$. We used the first $B = 5000$ Markov Chain Monte Carlo (MCMC) iterations as burn-in, and saved every 100th of the remaining iterations as part of thinning [89]. The “thinned” sequence contains 1000 iterations for each of the regression coefficients $\beta _j \ (j = 0, 1, 2, \ldots , p)$. For instance, Fig. F1 (in the supplementary file) shows the MCMC samples for different types of variables used in this study: Chittagong division (top-left), Wealth index: Poorest (top-right), Woman’s education: College or higher (bottom-left), and Woman’s age at marriage (bottom-right). It is evident that the chain has achieved stationarity after a few hundred’s of the iterations which justifies our choice for the burn-in of $B = 5000$.

We examined the auto-correlation function (ACF) against lag for the selected variables (Fig. F2 in the supplementary file). We determined that the successive iterations of the parameters from their respective posterior distribution were highly correlated which motivated us to save every 100th iterations (after burn-in) of the MCMC samples. The ACFs for these variables were rechecked after burn-in and thinning (figure is not shown), which showed negligible correlation between successive thinned iterations [68, 84]. The effective sample sizes for the thinned iterations after burn-in for Chittagong division, Wealth index: Poorest, Woman’s education: College or higher, and Woman’s age at marriage were 868, 888, 684 and 815, respectively. The ACFs and the effective sample sizes demonstrated the effectiveness of thinned iterations after burn-in.

## Results

The summary of the analytic sample is presented in Table 1 which shows the descriptive statistics for the variables used in this study. In summary, women studied had mean age 32.0 years with husband’s mean age 40.0 years, and were married at a mean age of 16.9 years. The averages of district-level adult literacy and contraceptive prevalence rates were $71.3\%$ and $63.5\%$, respectively. Of all the women, 17.9%, 25.8%, 43.4% and 13.0% had education preschool or none, elementary, high school, and college or higher, respectively. Sixty five percent of the women had media exposure, and about 97.8% of the women had a Bengali ethnicity. Approximately 41.8% of the women were from poorest and poorer households. Most women were from rural areas (80.3%), and one-fifth were from Dhaka division (20.2%).

**Table 1:** Descriptive statistics for the explanatory variables used in this study

Variables	Type	Mean	SD
Age at marriage	Quantitative	16.9	3.3
Age in years	Quantitative	32.0	8.7
Husband’s age	Quantitative	40.0	10.3
Adult literacy rate^a^	Quantitative	71.3	8.0
CPR^b^	Quantitative	63.5	9.9

Variables	Categories	Frequency	Percentage
Education	Preschool or none	9196	17.9
	Elementary	13242	25.8
	High school	22298	43.4
	College or Higher	6625	13.0
Media exposure	No	17848	34.8
	Yes	33513	65.3
Ethnicity	Others	1148	2.2
	Bengali	50213	97.8
Wealth index	Poorest	10720	20.9
	Poorer	10743	20.9
	Middle	10709	20.8
	Richer	10341	20.1
	Richest	8848	17.2
Area	Urban	10127	19.7
	Rural	41234	80.3
Division	Barishal	4541	8.8
	Chittagong	9141	17.8
	Dhaka	10367	20.2
	Khulna	8416	16.4
	Mymensingh	2670	5.2
	Rajshahi	6373	12.4
	Rangpur	6433	12.5
	Sylhet	3420	6.7

For the quantitative variables, we provide the mean and standard deviation (SD). For the categorical variables, we provide the frequency and percentage of cases within each category

^a^District level adult literacy rate

^b^District level contraceptive prevalence rate

Table 2 shows the posterior estimates and $95\%$ credible intervals (CI) of the incidence rate ratio (IRR) for the explanatory variables of the CEB among women of reproductive age for the unweighted and weighted Bayesian Poisson regression using the Normal prior. Tables S1 and S2 in the supplementary file show the corresponding estimates for the Laplace and Cauchy priors, respectively. The proposed weighted Bayesian Poisson regression models passed the overdispersion test [23]. The last row of Table 2 shows that the weighted regression model performed better than the unweighted model. Similar result followed (Tables S1 and S2) for the Laplace and Cauchy priors. The model with Normal prior was chosen for explaining the results based on the minimum WBIC.

**Fig. 3:**
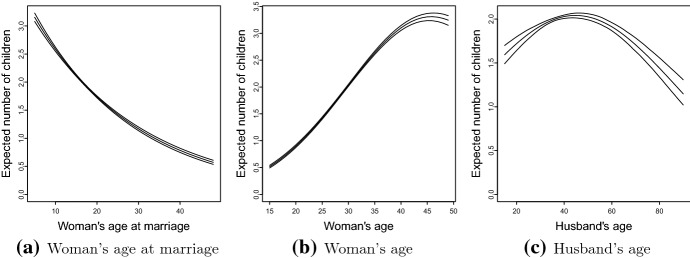
Estimates of expected number of children against woman’s age at marriage, woman’s age, and husband’s age

Results in Table 2 show that the woman’s age at marriage is significantly associated with expected CEB such that each one year increase in age at marriage was associated with $3.9\%$ decrease in mean CEB. Figure 3a shows the expected CEB with credible bands against woman’s age at marriage. The lower, second and third quartiles of woman’s age at marriage are 15, 16 and 18 (legal age at marriage) years, for which the expected CEB are 2.12, 2.04, and 1.88, respectively. As a realistic target to achieve for the age at marriage for the women in Bangladesh would be 20 years which would result in an expected CEB of 1.74 ($95\%$ CI $1.72-1.75$).

Based on exploratory analysis (Fig. F4a in the supplementary file), the quadratic effect of woman’s age on the number of CEB has been considered (Table 2). Figure 3b shows that with the increase of woman’ age the expected number of CEB increases which stabilizes (with increasing credible band) when the women reach 40 years and beyond. The expected number of CEB reaches its maximum 3.31 ($95\%$ CI $3.23-3.38$) when the women reach 46 years of age.

**Table 2:** Posterior estimates with $95\%$ credible intervals (CI) of the estimated coefficients (ln(IRR) - top part) and incidence rate ratio (IRR - bottom part) for the explanatory variables

Variables^a^	Categories/type	Unweighted			Weighted		
		ln(IRR)	$95\%$ CI		ln(IRR)	$95\%$ CI	
			LL	UL		LL	UL
Age at marriage	Quantitative	− 0.393	− 0.413	− 0.373	− 0.398	− 0.418	− 0.378
Age in years (L)	Quantitative	1.792	1.708	1.870	1.798	1.719	1.879
Age in years (Q)	Quantitative	− 0.020	− 0.021	− 0.018	− 0.020	− 0.021	− 0.019
Husband’s age (L)	Quantitative	0.244	0.188	0.307	0.252	0.186	0.314

Variables^a^	Categories/type	Unweighted			Weighted		
		IRR	$95\%$ CI		IRR	$95\%$ CI	
			LL	UL		LL	UL
Education	High school (ref)	$\ldots$	$\ldots$	$\ldots$	$\ldots$	$\ldots$	$\ldots$
	Preschool or none	1.084	1.065	1.103	1.086	1.068	1.105
	Elementary	1.052	1.038	1.067	1.053	1.039	1.066
	College or Higher	0.856	0.836	0.876	0.856	0.834	0.875
Media exposure	No (ref)	$\dots$	$\dots$	$\dots$	$\ldots$	$\ldots$	$\ldots$
	Yes	0.947	0.935	0.959	0.950	0.937	0.963
Ethnicity	Others (ref)	$\ldots$	$\ldots$	$\ldots$	$\ldots$	$\ldots$	$\ldots$
	Bengali	1.170	1.124	1.224	1.117	1.054	1.181
Wealth index	Middle (ref)	$\ldots$	$\ldots$	$\ldots$	$\ldots$	$\ldots$	$\ldots$
	Poorest	1.095	1.075	1.115	1.108	1.086	1.131
	Poorer	1.044	1.026	1.061	1.050	1.031	1.069
	Richer	0.971	0.952	0.989	0.970	0.953	0.988
	Richest	0.946	0.925	0.966	0.950	0.931	0.970
Area	Urban (ref)	$\ldots$	$\ldots$	$\ldots$	$\ldots$	$\ldots$	$\ldots$
	Rural	1.017	1.002	1.034	1.007	0.992	1.022
Division	Dhaka (ref)	$\ldots$	$\ldots$	$\ldots$	$\ldots$	$\ldots$	$\ldots$
	Barishal	1.034	1.006	1.066	1.047	1.014	1.080
	Chittagong	1.171	1.148	1.193	1.193	1.172	1.215
	Khulna	0.897	0.878	0.918	0.906	0.885	0.927
	Mymensingh	1.033	1.003	1.062	1.063	1.033	1.093
	Rajshahi	0.883	0.861	0.904	0.905	0.882	0.926
	Rangpur	0.982	0.959	1.008	0.989	0.960	1.017
	Sylhet	1.221	1.193	1.252	1.251	1.219	1.282
Adult literacy rate^b^	Quantitative	0.997	0.996	0.998	0.997	0.996	0.998
CPR^c^	Quantitative	0.997	0.996	0.998	0.997	0.996	0.998
WBIC^d^		151,184.41			150,048.51		

The lower and upper limits of the CIs are identified by LL and UL, respectively. The linear and quadratic terms for the age variables are denoted by L and Q, respectively, for which the coefficients are provided per 10 years. Here, we have used Normal prior distribution; and *ref* stands for the reference category

^a^Since there are both linear and quadratic terms in the model, the IRR is not meaningful and instead the coefficients from the models are reported. For interpretation of the effects, please see Fig. 3b and c

^b^District level adult literacy rate

^c^District level contraceptive prevalence rate

^d^Weighted Bayesian information criterion

Based on similar exploratory analysis (Fig. F4b in the supplement), the quadratic effect of husband’s age on the number of CEB has been considered (Table 2). Figure 3c shows that the expected number of children increases (with decreasing credible band) with the increase of husband’s age from 15 up to about 45 years, after which the number decreases (with increasing credible band). The expected number of CEB reaches its maximum 2.04 ($95\%$ CI $2.01-2.07$) when the husband’s age reaches 45 years.

**Fig. 4:**
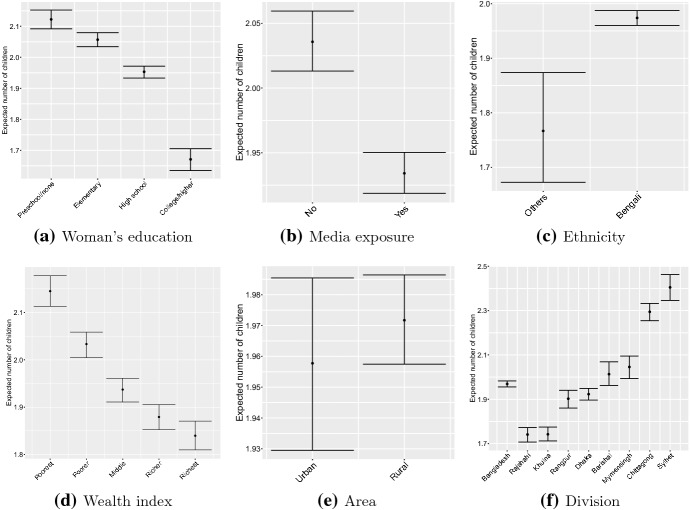
Estimates of expected number of children ever born against woman’s education, media exposure, ethnicity, wealth index, area of residence, and administrative division

The number of CEB was significantly associated with woman’s education. Women with education preschool or none and elementary have $8.6\%$ and $5.3\%$ higher number of CEB than the women with high school education, respectively. In contrast, women with college or higher education have $14.4\%$ lower number of CEB than the women with high school education (Table 2 and Fig. 4a). Women with high school education have expected number of CEB of 1.95 which is insignificantly lower than the national average (to be shown later). Women with education preschool or none have the highest number of CEB of 2.12, followed by women with elementary education with expected CEB of 2.06.

In this study, the media exposure of women was inversely associated with CEB (IRR 0.95; $95\%$ CI $0.94-0.96$), implying that women with media exposure, on average, have $5\%$ fewer number of CEB than the women without media exposure (Table 2 and Fig. 4b). Table 2 shows that the women with Bengali ethnicity have significantly higher number of CEB than the women with Others (non-Bengali) ethnicity (IRR 1.12; $95\%$ CI $1.05-1.18$). Figure 4c shows that the women with Bengali ethnicity have expected number of CEB of 1.97 compared to 1.77 of the women with non-Bengali ethnicity. The two CIs are non-overlapping with Bengali ethnicity showing very narrow width, which is due the larger sample size for the women with Bengali ethnicity (50,213) relative to that for the non-Bengali ethnicity (1,148).

Women in the categories poorest and poorer have $10.8\%$ and $5.0\%$ higher CEB than those in the middle income group, respectively. In contrast, the richer and richest have $3.0\%$ and $5.0\%$ lower CEB than the reference category, respectively. Figure 4d shows the expected number of CEB among women by their household wealth status. The women in the household with middle wealth status have expected number of CEB of 1.94 which is slightly (but insignificantly) lower than the national average (to be provided below). The women in the richer and the richest households have significantly lower expected number of CEB of 1.88 and 1.84, respectively, than the women in the households with middle wealth status. The women in the poorer and the poorest households have significantly larger expected number of CEB of 2.03 and 2.15, respectively, than the national average.

We did not find a statistically significant difference in mean number of CEB between rural and urban areas (Table 2, Fig. 4e). It is likely that the gap between the rural and urban areas is narrowing down over time.

At present Bangladesh is divided into eight administrative divisions: Barishal, Chittagong, Dhaka (holding nation’s capital city), Khulna, Mymensing, Rajshahi, Rangpur and Sylhet. Islam et al. [50] studied the differential of fertility by division and found marked variations in fertility, when Bangladesh was divided into six divisions only. Kabir et al. [55] studied the regional variation in fertility in Bangladesh using the the 2004 BDHS data. In this study, while the number of CEB in Rajshahi division is $9.5\%$ lower than the Dhaka division, the numbers of CEB in Chittagong and Sylhet divisions are $19.3\%$ and $25.1\%$ higher than the Dhaka division, respectively (Table 2). Figure 4f shows the expected number of CEB with $95\%$ CI in Bangladesh as a whole and by administrative divisions. The expected CEB in Bangladesh (national level) is 1.97 ($95\%$ CI $1.96-1.98$). The two divisions that have shown much improvement over the national average are Rajshahi and Khulna to which the expected number of CEB are 1.74 ($95\%$ CI $1.71-1.77$) and 1.74 ($95\%$ CI $1.71-1.78$), respectively. The two divisions that are doing worse in comparison to the national average are Chittagong and Sylhet with expected number of CEB 2.29 ($95\%$ CI $2.25-2.33$) and 2.41 ($95\%$ CI $2.35-2.46$), respectively.

**Fig. 5:**
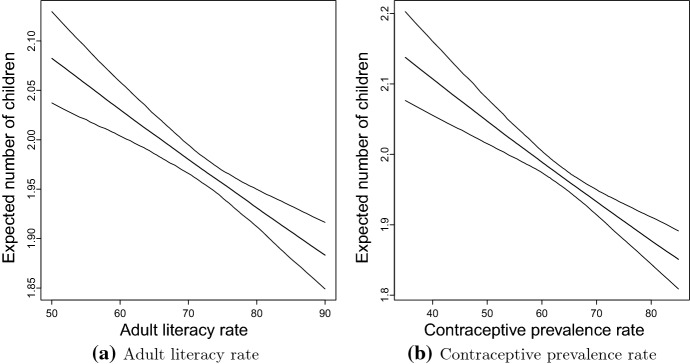
Estimates of expected number of children against district level adult literacy rate and contraceptive prevalence rate

In this study, effects of societal variables (characterised by district level compositional features) such as adult literacy and contraceptive prevalence rates on the number of CEB among married women reproductive age in Bangladesh have been studied for which the result follows. The district level adult literacy rate and CEB were significantly associated: For ten percent increase in the district level adult literacy rate, the number of CEB decreases by $3.0\%$ (Table 2). Figure 5a also shows that the association between expected number of CEB against district level adult literacy rate is negative. Note that the first, second (median) and third quartiles of adult literacy rates in Bangladesh are $67.5\%$, $71.5\%$ and $76.2\%$, respectively. When the district level adult literacy moves from the first, second to the third quartile, the expected number of CEB decreases from 1.99 ($95\%$ CI $1.98-2.01$) to 1.97 ($95\%$ CI $1.96-1.99$) to 1.95 ($95\%$ CI $1.93-1.96$). When the adult literacy rate moves up to $90\%$, the expected number of CEB decreases to 1.88 ($95\%$ CI $1.85-1.92$).

Similar to the effect of the district level adult literacy rate, the association between district level CPR on CEB is appeared to be significant: For ten percent increase of district level CPR, the number of CEB decreases by $3.0\%$ (Table 2). Figure 5b shows the expected number of CEB (with $95\%$ CI) against CPR. The relationship is negative: As the CPR goes up, the expected number of CEB goes down. Note that the first, second (median) and third quartiles of the district level CPRs in Bangladesh are $59.0\%$, $63.6\%$ and $69.9\%$, respectively. When the CPR goes up from the first, second to the third quartile, the expected number of CEB goes down from 2.00 ($95\%$ CI $1.98-2.01$) to 1.97 ($95\%$ CI $1.96-1.98$) to 1.93 ($95\%$ CI $1.92-1.95$). If the district level CPR could be pulled up to $80\%$, the expected number of CEB would decrease to 1.88 ($95\%$ CI $1.84-1.91$).

### The Effects of Weighting on Results

Tables 2, S1 and S2 contrast the results of weighted Poisson regression model with the unweighted model for the Normal, Laplace and Cauchy priors, respectively. The results were more-or-less comparable across the three tables (more details are in Sect. 5.2 below), and, therefore, we focused on the results from Normal prior (Table 2).

Some of the estimates and/or the confidence intervals of IRR were strikingly different across the weighted and unweighted models. For instance, the estimated coefficients for the rural area relative to urban area were 1.02 and 1.01 for the unweighted and weighted models, respectively. According to weighted model, the number of CEB in the rural area is insignificantly higher than the urban area since CIs are overlapping. On the other hand, the unweighted model shows that the CEB is significantly higher in the rural area than in the urban area. This is because, the unweighted model does not take into consideration the sampling weight for the women for which the average weight is higher in the urban area (1.15 versus 0.96).

As another example, the estimates of the IRRs for ethnicity using the unweighted and weighted models were 1.17 and 1.12, respectively, even though both estimates were statistically significant. The large differences in the estimates from the two models were due to larger mean sampling weights for the women with Bengali ethnicity of 1.01 than for the women of non-Bengali ethnicity of 0.48.

### Assessing Robustness of the Results

Following the idea presented in [25], the objective of this section is to show that the estimates of coefficients are robust against changing priors. Also, the second objective is to show that different types of priors can be applied to the proposed model. The fact that even with extreme priors such as Cauchy resulted in similar conclusions as other priors, the findings are robust against prior miss-specification.

Since there were barely any change for the factors age at marriage, woman’s age, husband’s age, woman’s education, media exposure, wealth index, area, adult literacy rate and CPR, we claim that the estimates of parameters in the proposed model are robust. However, we point out some negligible evidence of shrinkage for the weighted Poisson regression model reported across Tables 2, S1, and S2. For example, the estimated IRR for the Barishal division shrinks down from 1.05 to 1.05 to 1.04 for the Normal, Laplace and Cauchy priors, respectively. On the other hand, the estimates for the variable ‘ethnicity’ shrink down from 1.12 to 1.11 to 1.10 for the above three priors, respectively. The shrinkage by the stricter prior is evident especially when the variability of the response variable values between the categories of the explanatory variables is highly different. The results of small changes in the estimates are due to the large sample size of 51,361, given the fact that the posterior inference in Bayesian statistics is dominated by the data when the sample is large [32, 47]. In summary, it can be safely concluded that the estimates produced via the weighted Bayesian Poisson regression model are robust against changing priors [24].

## Discussion and Conclusion

This large-scale and nationally representative study suggests that woman’s fertility behavior is significantly associated with individual, household, and area-level factors in Bangladesh. After adjustment of sampling weight, the performance of the model, measured in terms of Weighted Bayesian Information Criterion, has been improved. The findings of this study suggest that existing family planning and reproductive health programs should be strengthened further in order to achieve the fertility target, with a focus not only on individual and household level factors, but also on area-level societal factors.

The observed association between decreased likelihood of CEB for increased age at marriage has been consistent with earlier studies [6, 43, 70, 80]. The results of this study reconfirm the direction of association of their findings. Women marrying at younger ages may have less schooling, awareness of family planning and decision-making abilities, which may contribute to higher fertility [69]. In this study, the observed median age at marriage for the women in Bangladesh is 16 years which yields an expected CEB of 2.04, a number higher than the national average. By increasing the age at marriage to the third quartile of 18 years (the legal age of marriage), the expected CEB can be decreased to 1.88. By increasing the woman’s age at marriage to 20 and 25 years, the government may further decrease the expected CEB to 1.74 and 1.42, respectively. When increasing the average age at marriage for women in Bangladesh to 25 years is highly ambitious, reaching that to 20 years is a reasonable target. Given that the country has been suffering from high child marriage [15, 56], the significant association of age at marriage with fertility highlights that programmatic efforts aimed at reducing woman’s fertility may need to be strengthened further. We also found that the ages of women and their spouse show quadratic relationship with CEB, implying that fertility rises with age and then declines after a certain age.

Education plays an important role in explaining CEB in Bangladesh. Educated women are more likely to have less expected number of CEB, which is supported by an earlier study on fertility in 38 developing countries including Bangladesh [100]. Similar inverse associations between woman’s education and fertility have been observed elsewhere [5, 8, 93]. In this study, women with preschool or no education have much higher expected number of CEB (2.12) than the women with college or higher education (1.67). Therefore, by educating women (from no education to the highest level) the government can curve down CEB by $21.3\%$. We note that the expected number of CEB for women with college education or higher is strikingly smaller than all of the other 3 categories of woman’s education and the national average as well. Possible reasons for the inverse association between woman’s education and CEB could be due to better knowledge of fertility and family planning, increased access to family planning options and practise of longer birth spacing among highly educated women [18, 60, 98, 100]. Strengthening educational initiatives for women, as well as empowering women, the government can aid in achieving the fertility goal.

It is well known that the links between education and fertility vary across countries and over time [13]. For example, the relationship between education and fertility in the Nordic countries has weakened over time [62] and appears to have disappeared [51]. In contrast, the findings of this study reveal that increased education of woman in the the developing countries is still very important to curve down fertility.

Moreover, women with exposure to the media have $5.0\%$ less expected number of CEB than the women without the media exposure. This findings are aligned with the results from earlier studies [28, 79]. Exposure to media may increase awareness of family planning and childcare among women, and, thus, contributing to lower fertility. Therefore, we recommend that the government bring more women under media exposure via mass digitization to the society to further reduce the fertility.

The differential of fertility among different ethnic groups had been studied in the United Kingdom by Coleman and Dubuc [21]. Their results showed that, while the total fertility in all ethnic groups had fallen from relatively high levels, the fertility rates among Bangladeshi women had been above the national average. Govindasamy and DaVanzo [41] also examined the impact of government policies on ethnic differences in fertility in Peninsular Malaysia. In our  study, ‘Bengali’ women have significantly higher expected number of CEB (1.97) than women who belongs to ‘Other’ ethnic group (1.77). The ethnic variation in the number of CEB in our study is supported by previous research in Bangladesh [26], which can be attributed to differences in socioeconomic status and socio-cultural norms between Bengali and Others.

Congruent with previous research [5, 61, 80], household wealth shows inverse relationship with number of CEB. When women from the richer households have far lower CEB ($\le 1.88$) than the national average, the women from the poorer households have very high CEB ($\ge 2.03$). Wealth-related variation may be related to affluent women’s greater access to health care and family planning options (e.g., contraceptive use). While there has been a lack of association between wealth and fertility in contemporary industrialized populations [94], our study reveals that wealth is still an important determinant of fertility in Bangladesh. Therefore, policymakers may provide more attention to the women from the poorer households, and the utmost level of attention to the women from the poorest households. The government may opt to include the socioeconomically disadvantaged women under social-safety-net, and increase vigilance to improve conditions of their access to higher education.

The urban and rural differential of fertility has been a widely studied factor in Bangladesh [48, 57, 58], where most of the studies revealed higher fertility in the rural area. In this study, The CIs for the place of residence overlapped in the weighted model, implying that there is non-significant difference in CEB between women from rural and urban areas. This suggests that the differences in CEBs between rural and urban areas are shrinking over time. Interestingly, the Bayesian unweighted Poisson regression implied that the expected CEB is still significantly higher in the rural area than the urban area in Bangladesh. From analytical point of view, this could be because the Bayesian model applies more shrinkage when there is larger difference in group sizes. The sizes for the rural and urban areas are 41,234 and 10,127, respectively. The effect of smaller group size is reflected in the wider CI of the number of CEB for the urban dwelling women. From social perspective, the observed overlapping/shrinking differences of expected number of CEB in the rural and urban areas from the weighted model could be attributed to the rapid urbanization and digitization of the society.

Individual and household level variables only tell part of the narrative of fertility decline, and studies that focus solely on them are more likely to ignore the impact of group-level factors (i.e. community or neighbourhood). Our study suggested that district-level social environmental features (such as adult literacy and contraceptive prevalence rates) are inversely related to the number of CEB. Women who live in communities with a higher literacy rate may have better access to health care, greater information and awareness about family planning, and higher autonomy in decision-making [101], all of which may lead to a lower fertility rate. Area-specific literacy rate not only reveals how a population’s resources are distributed, but it also has the potential to influence crucial social dynamics [75]. Once a critical mass of literate people has been reached, social interaction between literate and illiterate people accelerates the pace of fertility drop. An increased frequency of interaction with more literate people could be the key driver of this association. Furthermore, the adoption of low fertility norms and behaviour may be the outcome of a population-wide copying mechanism. People in highly educated societies are likely to interact with and witness the reproductive behaviour of literate people in their societies (e.g., neighbours and friends), who may favour lower fertility [72]. Another factor that may contribute to lower fertility in communities with higher literacy rates is the lower rate of early marriage and childbearing [36] and higher rates of contraceptive use [40]. A community with a higher percentage of contraception use may have greater accessibility and acceptability of family planning programs for a larger group of people, lowering the expected CEB further.

This research provides a foundation for the need of incorporating district-level social environmental features in planning fertility control strategies (e.g. community-based intervention and resource allocation). Implementing educational initiatives at the local level has the potential to educate residents and change community norms regarding family planning, which could have a positive impact on fertility reduction. By increasing awareness and access to services, increased education can enhance contraceptive use, thereby contributing to fertility reduction [64]. Policymakers may choose to intervene in areas with low literacy and low contraceptive uptake in order to reduce fertility [50]. These findings also highlight the importance of further research into other social and environmental features to understand the underlying mechanism of fertility. However, we note that district is a broad administrative boundary (see Fig. F3a), there is likely to be greater heterogeneity in health behaviour among individuals within a district, and thus it may not adequately capture social dynamics. As a result, we propose using smaller area characteristics (such as sub-district, union, and village) as societal variables in future research.

The results of this study show that the expected number of birth per woman in Bangladesh has been declined from 6.95 in 1970 and 2.06 in 2017 to 1.97 in 2019. The two administrative divisions (Chittagong with mean CEB 2.29 and Sylhet with 2.41) are performing very poorly relative to the national average. In contrast, the Khulna and Rajshahi divisions outperform the other divisions as well as the national average. These findings are supported by earlier study [50]. To further reduce the CEB, the government, NGOs, and policymakers may need to pay more attention to trailing divisions (Chittagong and Sylhet). Islam et al. [50] reported that ages at first marriage and first birth were the lowest in Khulna and Rajshahi division which along with higher rate of use of family planning methods, low level of desired fertility and longer non-first birth interval may have contributed to lower fertility rates in these divisions. Such comparative features contributing to high and low expected CEBs in these divisions can help policymakers develop targeted policies to lower overall CEBs.

From methodological perspective, Hastie and Tibshirani, Fortheringham et al. [44, 29] and Nakaya et al. [81] proposed geographically weighted Poisson regression (GWPR) by incorporating geographically defined weight function to estimate spatial variation in the model parameters. In GWPR, which is applied to spatial data, the weights are estimated using a kernel function of the geographic locations obtained in terms of latitude and longitude. In our study, the individual level data provided by [10, 38] are non-spatial, and, therefore, the application of GWPR may not be feasible.

The 2019 MICS survey [38] had emphasized on the use of survey weights in model building to ensure generalizability of the results. Therefore, following Newton and Raftery [83] and Wang [99], we incorporated these weights in the likelihood function construction. Different types of priors, designed from the perspective of imposing shrinkage, have been explored to demonstrate flexibility of the proposed methodology and robustness of the results. The posterior distribution for the parameter has been derived, and the Metropolis algorithm to generate posterior values has been presented. To compare models, the Weighted Bayesian Information Criterion has been proposed which simplifies to a regular BIC when the survey weight is one (i.e., equal weight) for all subjects. A possible future research of this methodology would be extending the weighted model by incorporating the multilevel models [39] by employing the hierarchical Poisson regression approach [19].

Overall, this study quantified the exposition of different factors operating at individual, household, and area-levels with fertility behaviour of women in Bangladesh. The key factors are age at marriage, ages of woman and spouse, education, media exposure, household wealth, place of residence, district level adult literacy rate, contraceptive use rate, and administrative divisions. Since Bangladesh may need to further reduce its fertility rate, it is important that policymakers continue to support expenditure in educational programs, increase awareness to family planning, and enhance access to family planning services. In addition, stronger mass media campaign promoting the benefits of family planning may result in a shift in attitudes against large families. Identifying geographical regions to prioritize intervention is also important for the government and non-government organizations.

This study had a few limitations. A cause-and-effect link between outcome and exposure or covariates could not be established owing to the cross-sectional nature of the data. There may be recall or social desirability bias during the data collection process; however, this is a common problem in survey data, and survey administrators always strive to reduce these biases, making them less likely to influence overall findings. We did not have access to several other variables (e.g., women’s beliefs, access to health care, and community-based family planning workers) which may be important for policy formation and warrant further investigation. Despite these limitations, the MICS data from Bangladesh represents a large survey sample that can be utilised to evaluate fertility patterns and associated factors.

## Supplementary Information

Below is the link to the electronic supplementary material.

Supplementary file1 (PDF 5487 KB)

## Data Availability

Data are publicly available from Bangladesh Multiple Indicator Cluster Survey of 2019 and Bangladesh Sample Vital Registration System of 2018. The computer codes are available at https://github.com/jhtomal/JSTA.git.

## Abbreviations

ACF: Auto-correlation function
AIC: Akaike Information criterion
BBS: Bangladesh Bureau of Statistics
BIC: Bayesian information criterion
CEB: Number of children ever born
CI: Credible interval
CPR: Contraceptive prevalence rate
GFR: General fertility rate
GOB: Government of Bangladesh
HPSNP: Health, Population and Nutrition Sector Program
IRR: Incidence rate ratio
MCMC: Markov Chain Monte Carlo
MICS: Multiple indicator cluster survey
SD: Standard deviation
SVRS: Sample vital registration system
TFR: Total fertility rate
UNICEF: United Nations Children’s Fund
WBIC: Weighted Bayesian Information Criterion

## Footnotes

1 https://extranet.who.int/ncdccs/Data/BGD_B3_OP_NCDC_04 May 2017.docx.

## References

[1] Ahmed B (1981). Differential fertility in Bangladesh: a path analysis. Social Biol..

[2] Akaike H (1974). A new look at the statistical model identification. IEEE Trans. Automat. Control.

[3] Akaike H (1979). A Bayesian extension of the minimum AIC procedure of autoregressive model fitting. Biometrika.

[4] Al-Awadhi F, Hurn M, Jennison C (2004). Improving the acceptance rate of reversible jump MCMC proposals. Stat. Prob. Lett..

[5] Amara M (2015). Multilevel modelling of individual fertility decisions in Tunisia: household and regional contextual effect. Social Indicators Res..

[6] Ariho P, Kabagenyi A, Nzabona A (2018). Determinants of change in fertility pattern among women in Uganda during the period. Fertility Res. Pract..

[7] Ariho P, Nzabona A (2019). Determinants of change in fertility among women in rural areas of Uganda. J. Pregnancy.

[8] Arokiasamy P, McNay K, Cassen RH (2004). Female education and fertility decline: recent developments in the relationship. Econ. Polit. Weekly.

[9] Banner KM, Irvine KM, Rodhouse TJ (2020). The use of Bayesian priors in ecology: the good, the bad and the not great. Methods Ecol. Evol..

[10] BBS (2019). Report on Bangladesh Sample Vital Statistics 2018.

[11] Bedrick EJ, Christensen R, Johnson W (1996). A new perspective on priors for generalized linear models. J. Am. Stat. Assoc..

[12] Bergaglio M (2017). The contemporary illusion: population growth and sustainability. Environ. Dev. Sustain..

[13] Bijlsma MJ, Wilson B (2020). Modelling the socio-economic determinants of fertility: a mediation analysis using the parametric g-formula. J. R Stat. Soc..

[14] Bongaarts J (2009). Human population growth and the demographic transition. Philos. Trans. R Soc. B Biol. Sci..

[15] Caldwell BK (2005). Factors affecting female age at marriage in South Asia. Asian Popul. Stud..

[16] Cameron, A. C., Trivedi, P. K. : *Regression Analysis of Count Data*. Econometric Society Monographs. Cambridge University Press, 2 edn (2013)

[17] Chakrabarti, A., Ghosh, J. K.: AIC, BIC and recent advances in model selection. In *Philosophy of Statistics* (eds. P. S. Bandyopadhyay and M. R. Forster), vol. 7 of *Handbook of the Philosophy of Science*, 583–605. Amsterdam: North-Holland. https://www.sciencedirect.com/science/article/pii/B9780444518620500186 (2011)

[18] Chaudhury RH (1984). The influence of female education, labor force participation, and age at marriage on fertility behavior in Bangladesh. Social Biol..

[19] Christiansen CL, Morris CN (1997). Hierarchical Poisson regression modeling. J. Am. Stat. Assoc..

[20] Cohen JE (2003). Human population: the next half century. Science.

[21] Coleman DA, Dubuc S (2010). The fertility of ethnic minorities in the UK, 1960s–2006. Popul. Stud..

[22] Consul P, Famoye F (1992). Generalized Poisson regression model. Commun. Stat. Theory Methods.

[23] Dean C, Lawless JF (1989). Tests for detecting overdispersion in Poisson regression models. J. Am. Stat. Assoc..

[24] Dorsett R (2021). A Bayesian structural time series analysis of the effect of basic income on crime: evidence from the Alaska Permanent Fund. J. R Stat. Soc. A (Statistics in Society).

[25] Doucouliagos C, Hennessy J, Mallick D (2021). Health aid, governance and infant mortality. J. R Stat. Soc. A (Statistics in Society).

[26] DPSDU and BBS *Fertility Differentials in Bangladesh: Trends and Determinants*. Bangladesh Bureau of Statistics (2015)

[27] El-Sayyad GM (1973). Bayesian and classical analysis of Poisson regression. J. R Stat. Soc. B (Methodological).

[28] Fazle Rabbi AM (2012). Mass media exposure and its impact on fertility: current scenario of Bangladesh. J. Sci. Res..

[29] Fotheringham, S., Brunsdon, C., Charlton, M.: *Geographically Weighted Regression: The Analysis of Spatially Varying Relationships*. Wiley (2002)

[30] Frome EL (1983). The analysis of rates using Poisson regression models. Biometrics.

[31] Gelfand AE (2000). Gibbs sampling. J. Am. Stat. Assoc..

[32] Gelman, A., Carlin, J., Stern, H., Dunson, D., Vehtari, A., Rubin, D.: *Bayesian Data Analysis, Third Edition*. Chapman & Hall/CRC Texts in Statistical Science. Taylor & Francis. https://books.google.ca/books?id=ZXL6AQAAQBAJ (2013)

[33] Gerland P, Raftery AE, Ševčíková H, Li N, Gu D, Spoorenberg T, Alkema L, Fosdick BK, Chunn J, Lalic N, Bay G, Buettner T, Heilig GK, Wilmoth J (2014). World population stabilization unlikely this century. Science.

[34] Geyer CJ (1992). Practical Markov Chain Monte Carlo. Stat. Sci..

[35] Ghosh J, Li Y, Mitra R (2018). On the use of Cauchy prior distributions for Bayesian logistic regression. Bayesian Anal..

[36] Glick P, Handy C, Sahn DE (2015). Schooling, marriage, and age at first birth in Madagascar. Popul. Stud..

[37] GOB (2017) *4th Health, Population and Nutrition Sector Programmme (4th HPNSP)*. Dhaka, Bangladesh: Hospital Services Management, Ministry of Health and Family Welfare, Government of the People’s Republic Of Bangladesh

[38] GOB, BBS and UNICEF (2019) *Bangladesh Multiple Indicator Cluster Survey 2019 Survey Findings Report*. Dhaka, Bangladesh: Government of the People’s Republic Of Bangladesh, Bangladesh Bureau of Statistics, and United Nations Children’s Fund

[39] Goldstein, H. : *Multilevel Statistical Models*. Wiley Series in Probability and Statistics (2010)

[40] Goni A, Rahman M (2012). The impact of education and media on contraceptive use in bangladesh: a multivariate analysis. Int. J. Nurs. Pract..

[41] Govindasamy P, DaVanzo J (1992). Ethnicity and fertility differentials in peninsular Malaysia: do policies matter?. Popul. Dev. Rev..

[42] Hahn Y, Islam A, Nuzhat K, Smyth R, Yang H-S (2018). Education, marriage and fertility: Long-term evidence from a female stipend program in Bangladesh. Econ. Dev. Cult. Change.

[43] Harwood-Lejeune A (2001). Rising age at marriage and fertility in Southern and Eastern Africa. Eur. J. Popul..

[44] Hastie T, Tibshirani R (1993). Varying-coefficient models. J. R. Stat. Soc. B (Methodological).

[45] Hastings WK (1970). Monte Carlo sampling methods using Markov chains and their applications. Biometrika.

[46] Hoerl AE, Kennard RW (1970). Ridge regression: biased estimation for nonorthogonal problems. Technometrics.

[47] Hoff, P. D. : Nonconjugate priors and Metropolis-Hastings algorithms. In *A First Course in Bayesian Statistical Methods*, 171–193. New York, NY: Springer New York. 10.1007/978-0-387-92407-6_10 (2009)

[48] Hoque MN, Murdock SH (1997). Socioeconomic development, status of women, family planning, and fertility in Bangladesh: a district level analysis. Social Biol..

[49] Ingrassia S, Punzo A, Vittadini G, Minotti SC (2015). Erratum to: The generalized linear mixed cluster-weighted model. J. Classification.

[50] Islam MM, Rob U, Chakroborty N (2003). Regional variations in fertility in Bangladesh. Genus.

[51] Jalovaara M, Neyer G, Andersson G, Dahlberg J, Dommermuth L, Fallesen P, Lappegård T (2019). Education, gender, and cohort fertility in the Nordic countries. Eur. J. Popul..

[52] Jeffreys H (1946). An invariant form for the prior probability in estimation problems. Proc. R Soc. London A Math. Phys. Sci..

[53] Johnson, N. L., Kemp, A. W., Kotz, S.: *Poisson Distribution*, 156–207. John Wiley & Sons, Ltd. https://onlinelibrary.wiley.com/doi/abs/10.1002/0471715816.ch4 (2005)

[54] Jolicoeur, P. : The Poisson distribution. In *Introduction to Biometry*, 124–133. Boston, MA: Springer US. 10.1007/978-1-4615-4777-8_19 (1999)

[55] Kabir A, Ali R, Islam MS, Kawsar LA, Islam MA (2009). A comparison of regional variations of fertility in Bangladesh. Int. Quat. Commun. Health Educ..

[56] Kamal SMM, Hassan CH, Alam GM, Ying Y (2015). Child marriage in Bangladesh: trends and determinants. J. Biosoc. Sc..

[57] Khan H, Raeside R (1997). Factors affecting the most recent fertility rates in urban-rural Bangladesh. Social Sci. Med..

[58] Khan HTA, Raeside R (1994). Urban and rural fertility in Bangladesh: a causal approach. Social Biol..

[59] Kim S, Chen Z, Zhang Z, Simons-Morton BG, Albert PS (2013). Bayesian Hierarchical Poisson regression models: an application to a driving study with kinematic events. J. Am. Stat. Assoc..

[60] King, E. M., Hill, M. A.: *Women’s education in developing countries*. The World Bank. https://elibrary.worldbank.org/doi/abs/10.1596/0-8018-4534-3 (1993)

[61] Kiser H, Hossain MA (2019). Estimation of number of ever born children using zero truncated count model: evidence from Bangladesh Demographic and Health Survey. Health Info. Sci. Syst..

[62] Øystein K, Rindfuss RR (2008). Changing relationships between education and fertility: a study of women and men born 1940 to 1964. Am. Sociol. Rev..

[63] Lambert D (1992). Zero-inflated Poisson regression, with an application to defects in manufacturing. Technometrics.

[64] Latif MA (1994). Programme impact on current contraception in Bangladesh. Bangladesh Dev. Stud..

[65] Lawless JF (1987). Negative Binomial and mixed Poisson regression. Canad. J. Stat..

[66] Lemoine NP (2019). Moving beyond noninformative priors: why and how to choose weakly informative priors in Bayesian analyses. Oikos.

[67] Lenk PJ (1988). The Logistic Normal distribution for Bayesian, nonparametric, predictive densities. J. Am. Stat. Assoc..

[68] Link WA, Eaton MJ (2012). On thinning of chains in MCMC. Methods Ecol. Evol..

[69] Mahmood N, Ringheim K (1997). Knowledge, approval and communication about family planning as correlates of desired fertility among spouses in Pakistan. Int. Family Plan. Perspect..

[70] Malaker CR (1972). Female age at marriage and the birth rate in India. Social Biol..

[71] Max Roser, H. R., Ortiz-Ospina, E.: World population growth. *Our World in Data*. Https://ourworldindata.org/world-population-growth (2013)

[72] McNay K, Arokiasamy P, Cassen R (2003). Why are uneducated women in India using contraception? A multilevel analysis. Popul. Stud..

[73] Metropolis N, Rosenbluth AW, Rosenbluth MN, Teller AH, Teller E (1953). Equation of state calculations by fast computing machines. J. Chem. Phys..

[74] Metropolis N, Ulam S (1949). The Monte Carlo method. J. Am. Stat. Assoc..

[75] Mezirow, J. : Educating adults in family planning. *World Education Issues* (1972)

[76] Miah MMR (1993). Determinants of high fertility in Bangladesh: their implications for social development. Int. Rev. Modern Sociol..

[77] Mitchell TJ, Beauchamp JJ (1988). Bayesian variable selection in linear regression. J. Am. Stat. Assoc..

[78] Myburgh CAL (1956). Estimating the fertility and mortality of African populations from the total number of children ever born and the number of these still living. Popul. Stud..

[79] Nahar MZ, Zahangir MS (2019). Determinants of fertility in Bangladesh: evidence from the 2014 Demographic and Health Survey. Int. Quart. Commun. Health Educ..

[80] Nahar MZ, Zahangir MS, Islam SS (2013). Age at first marriage and its relation to fertility in Bangladesh. Chinese J. Popul. Resour. Environ..

[81] Nakaya T, Fotheringham AS, Brunsdon C, Charlton M (2005). Geographically weighted Poisson regression for disease association mapping. Stat. Med..

[82] Neath AA, Cavanaugh JE (2012). The Bayesian information criterion: background, derivation, and applications. WIREs Comput. Stat..

[83] Newton MA, Raftery AE (1994). Approximate Bayesian inference with the weighted likelihood bootstrap. J. R. Stat. Soc. B (Methodological).

[84] Owen AB (2017). Statistically efficient thinning of a Markov Chain Sampler. J. Comput. Graph. Stat..

[85] Pan W (2001). Akaike’s information criterion in generalized estimating equations. Biometrics.

[86] Polson NG, Sokolov V (2019). Bayesian regularization: from Tikhonov to horseshoe. WIREs Comput. Stat..

[87] Poston DL, Bouvier LF (2010). Population and society: an introduction to demography.

[88] R Core Team (2022) *R: A Language and Environment for Statistical Computing*. R Foundation for Statistical Computing, Vienna, Austria. https://www.R-project.org

[89] Riabiz, M., Chen, W., Cockayne, J., Swietach, P., Niederer, S. A., Mackey, L., Oates, C. J.: Optimal thinning of MCMC output. https://arxiv.org/abs/2005.03952 (2020)

[90] Ribeiro MC, Sousa AJ, Pereira MJ (2016). A coregionalization model can assist specification of Geographically Weighted Poisson Regression: application to an ecological study. Spat. Spatio-temp. Epidemiol..

[91] Robert CP, Chopin N, Rousseau J (2009). Rejoinder: Harold Jeffreys’s theory of probability revisited. Stat. Sci..

[92] Schwarz G (1978). Estimating the dimension of a model. Ann. Stat..

[93] Snopkowski K, Towner MC, Shenk MK, Colleran H (2016). Pathways from education to fertility decline: a multi-site comparative study. Philos. Trans. R. Soc. B: Biol. Sci..

[94] Stulp G, Barrett L (2016). Wealth, fertility and adaptive behaviour in industrial populations. Philos. Trans. R. Soc. B: Biol. Sci..

[95] Tibshirani R (1996). Regression shrinkage and selection via the Lasso. J. R. Stat. Soc. Series B (Methodological).

[96] Tsionas EG (2001). Bayesian multivariate Poisson regression. Commun. Stat. - Theory and Methods.

[97] United Nations (2020) World fertility and family planning 2020: Highlights. https://www.un.org/development/desa/pd/content/world-fertility-and-family-planning-2020-highlights. United Nations Department of Economic and Social Affairs

[98] Upadhyay UD, Gipson JD, Withers M, Lewis S, Ciaraldi EJ, Fraser A, Huchko MJ, Prata N (2014). Women’s empowerment and fertility: a review of the literature. Social Sci. Med..

[99] Wang, S. X.: *Maximum weighted likelihood estimation*. Ph.D. thesis, Department of Statistics, University of British Columbia, Vancouver, BC, Canada. https://open.library.ubc.ca/collections/ubctheses/831/items/1.0090880 (2001)

[100] Weinberger MB (1987). The relationship between women’s education and fertility: selected findings from the world fertility surveys. Int. Family Plan. Perspect..

[101] Yaya S, Bishwajit G, Ekholuenetale M, Shah V (2017). Awareness and utilization of community clinic services among women in rural areas in Bangladesh: a cross-sectional study. PLOS ONE.

[102] Zhang Y, Li Y, Deng W, Huang K, Yang C (2021). Complex networks identification using Bayesian model with independent Laplace prior. Chaos Interdiscip. J. Nonlinear Sci..

