# Supplementary material for: Weighted Bayesian Poisson Regression for The Number of Children Ever Born per Woman in Bangladesh
Source: J Stat Theory Appl. 2022 Jun 14;21(3):79–105. doi: 10.1007/s44199-022-00044-2 (PMC9388455; doi:10.1007/s44199-022-00044-2)
Supplement: Supplementary file 1 — Supplementary file1 (PDF 5487 KB) [file 44199_2022_44_MOESM1_ESM.pdf]

Supplementary materials

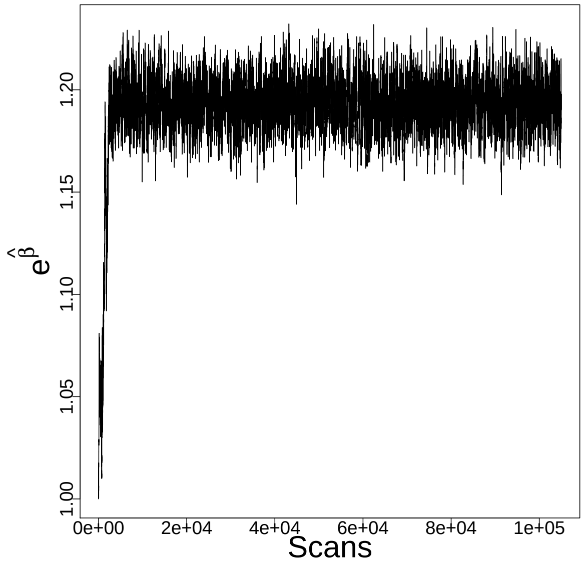

(a) Division: Chittagong

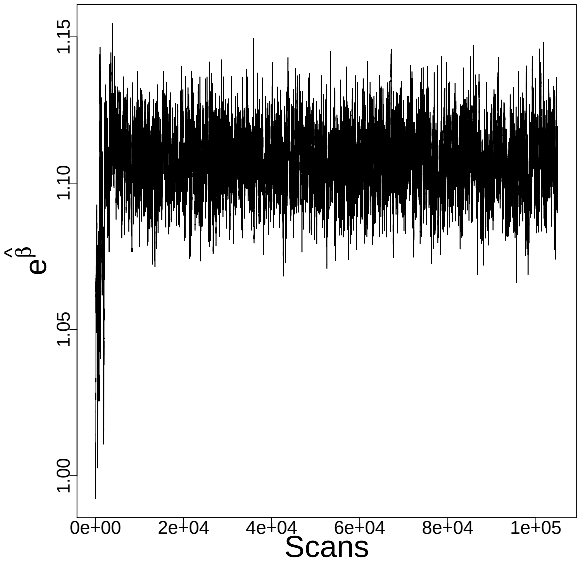

(b) Wealth index: Poorest

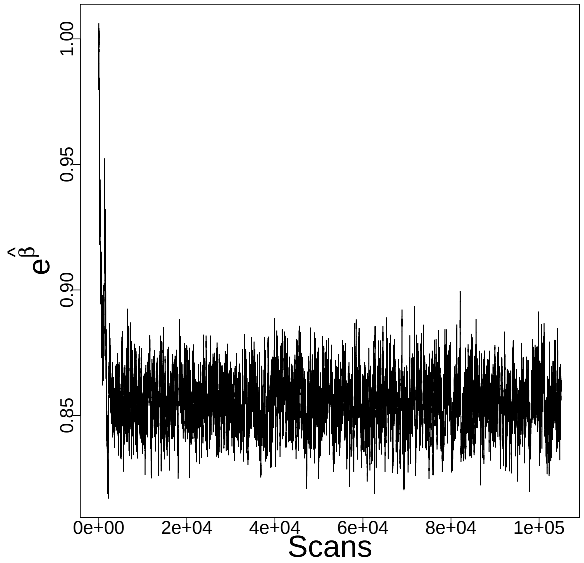

(c) Woman's education: College or higher

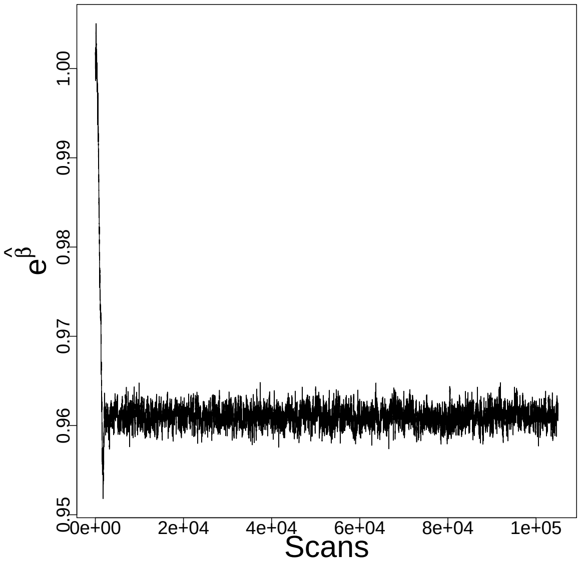

(d) Woman's age in years at marriage

Figure F1. Markov Chain Monte Carlo (MCMC) samples against iteration.

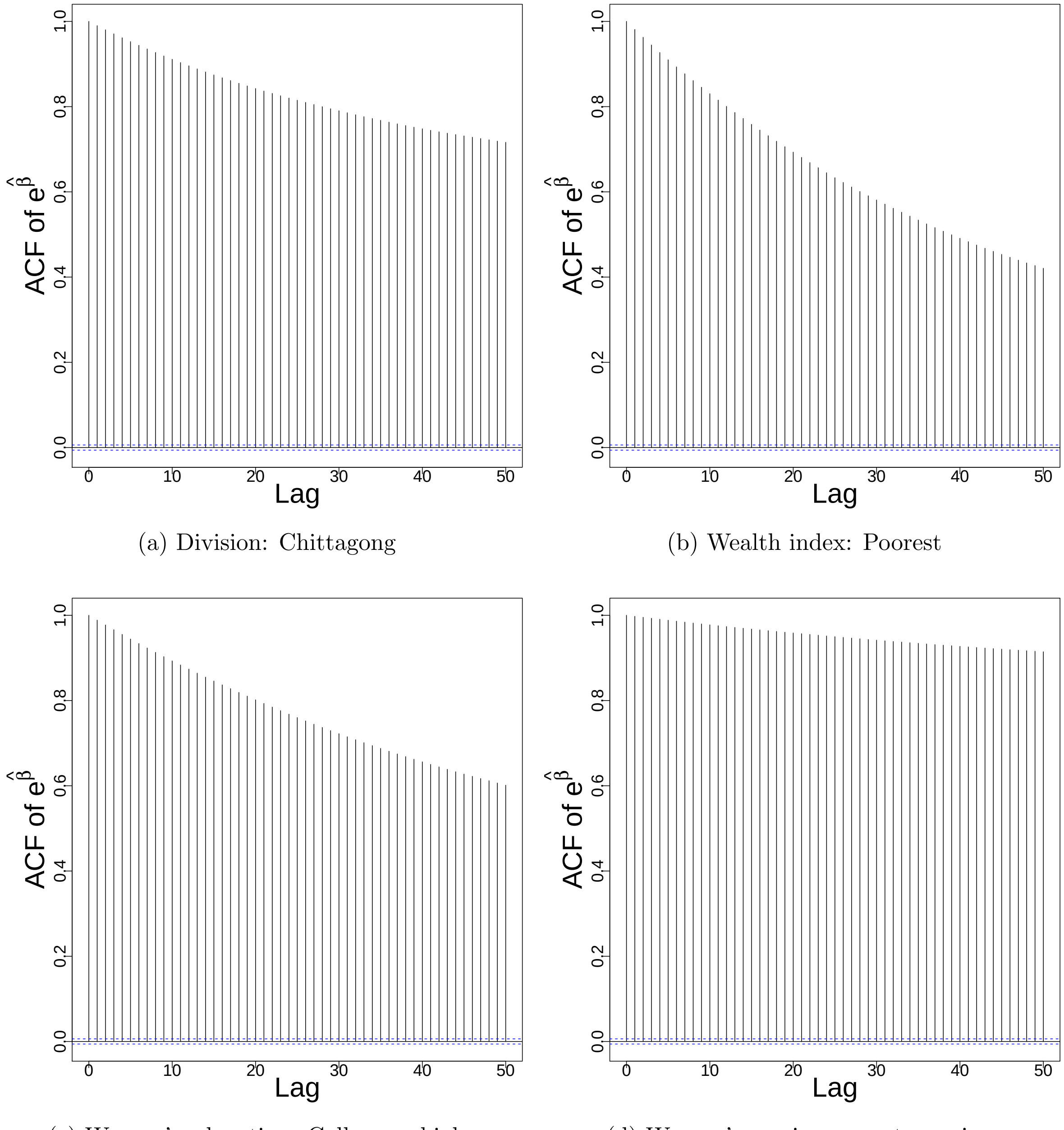

(a) Division: Chittagong

(b) Wealth index: Poorest

(c) Woman's education: College or higher

(d) Woman's age in years at marriage

Figure F2. Auto-correlation functions (ACF) against lag.

Table S1. Posterior estimates with 95% credible intervals (CI) of the estimated coefficients (ln(IRR) - top part) and incidence rate ratio (IRR - bottom part) for the explanatory variables. The lower and upper limits of the CIs are identified by LL and UL, respectively. The linear and quadratic terms for the age variables are denoted by L and Q, respectively, for which the coefficients are provided per 10 years. Here, we have used Laplace prior distribution; and *ref* stands for the reference category.

Variables‖	Categories/type	Unweighted			Weighted		
		ln(IRR)	95% CI		ln(IRR)	95% CI	
			LL	UL		LL	UL
Age at marriage	Quantitative	-0.393	-0.414	-0.374	-0.398	-0.419	-0.378
Age in years (L)	Quantitative	1.791	1.707	1.87	1.799	1.716	1.878
Age in years (Q)	Quantitative	-0.020	-0.021	-0.018	-0.020	-0.021	-0.019
Husband's age (L)	Quantitative	0.244	0.180	0.304	0.250	0.189	0.314
Husband's age (Q)	Quantitative	-0.003	-0.003	-0.002	-0.003	-0.003	-0.002
		IRR	95% CI		IRR	95% CI	
			LL	UL		LL	UL
Education	High school (ref)	···	···	···	···	···	···
	Preschool or none	1.083	1.064	1.102	1.086	1.067	1.104
	Elementary	1.052	1.037	1.067	1.053	1.038	1.068
	College or Higher	0.856	0.835	0.876	0.855	0.834	0.875
Media exposure	No (ref)	···	···	···	···	···	···
	Yes	0.946	0.934	0.959	0.951	0.937	0.964
Ethnicity	Others (ref)	···	···	···	···	···	···
	Bengali	1.167	1.121	1.217	1.112	1.056	1.178
Wealth index	Middle (ref)	···	···	···	···	···	···
	Poorest	1.095	1.076	1.116	1.107	1.086	1.129
	Poor	1.043	1.024	1.061	1.050	1.030	1.067
	Rich	0.971	0.955	0.988	0.970	0.951	0.990
	Richest	0.946	0.926	0.966	0.950	0.929	0.970
Area	Urban (ref)	···	···	···	···	···	···
	Rural	1.017	1.001	1.034	1.007	0.992	1.023
Division	Dhaka (ref)	···	···	···	···	···	···
	Barishal	1.033	1.004	1.062	1.045	1.014	1.079
	Chittagong	1.170	1.148	1.194	1.192	1.172	1.214
	Khulna	0.898	0.879	0.918	0.906	0.883	0.927
	Mymensingh	1.032	1.001	1.062	1.063	1.033	1.091
	Rajshahi	0.884	0.862	0.905	0.906	0.882	0.928
	Rangpur	0.984	0.959	1.008	0.990	0.964	1.015
	Sylhet	1.222	1.193	1.253	1.251	1.219	1.285
Adult literacy rate‡	Quantitative	0.997	0.996	0.998	0.998	0.997	0.998
CPR§	Quantitative	0.997	0.996	0.998	0.997	0.996	0.998
WBIC¶		151,184.55			150,048.67		

Table S2. Posterior estimates with 95% credible intervals (CI) of the estimated coefficients (ln(IRR) - top part) and incidence rate ratio (IRR - bottom part) for the explanatory variables. The lower and upper limits of the CIs are identified by LL and UL, respectively. The linear and quadratic terms for the age variables are denoted by L and Q, respectively, for which the coefficients are provided per 10 years. Here, we have used Cauchy prior distribution; and *ref* stands for the reference category.

Variables‖	Categories/type	Unweighted			Weighted		
		ln(IRR)	95% CI		ln(IRR)	95% CI	
			LL	UL		LL	UL
Age at marriage	Quantitative	-0.394	-0.414	-0.376	-0.398	-0.417	-0.377
Age in years (L)	Quantitative	1.796	1.713	1.872	1.804	1.720	1.881
Age in years (Q)	Quantitative	-0.020	-0.021	-0.019	-0.020	-0.021	-0.019
Husband's age (L)	Quantitative	0.238	0.176	0.302	0.243	0.182	0.308
Husband's age (Q)	Quantitative	-0.003	-0.003	-0.002	-0.003	-0.003	-0.002
		IRR	95% CI		IRR	95% CI	
			LL	UL		LL	UL
Education	High school (ref)	···	···	···	···	···	···
	Preschool or none	1.082	1.062	1.100	1.085	1.065	1.106
	Elementary	1.050	1.035	1.065	1.052	1.036	1.066
	College or Higher	0.855	0.833	0.877	0.855	0.835	0.875
Media exposure	No (ref)	···	···	···	···	···	···
	Yes	0.947	0.933	0.960	0.950	0.936	0.964
Ethnicity	Others (ref)	···	···	···	···	···	···
	Bengali	1.168	1.123	1.217	1.103	1.039	1.176
Wealth index	Middle (ref)	···	···	···	···	···	···
	Poorest	1.096	1.076	1.118	1.109	1.087	1.132
	Poor	1.043	1.024	1.063	1.050	1.030	1.069
	Rich	0.974	0.956	0.992	0.973	0.955	0.993
	Richest	0.948	0.927	0.968	0.953	0.932	0.974
Area	Urban (ref)	···	···	···	···	···	···
	Rural	1.016	1.000	1.033	1.007	0.992	1.021
Division	Dhaka (ref)	···	···	···	···	···	···
	Barishal	1.029	1.002	1.060	1.042	1.009	1.077
	Chittagong	1.169	1.146	1.191	1.192	1.170	1.213
	Khulna	0.897	0.878	0.917	0.907	0.883	0.929
	Mymensingh	1.025	0.999	1.056	1.062	1.029	1.089
	Rajshahi	0.883	0.863	0.904	0.906	0.884	0.927
	Rangpur	0.985	0.959	1.007	0.992	0.968	1.013
	Sylhet	1.221	1.190	1.253	1.252	1.220	1.284
Adult literacy rate‡	Quantitative	0.997	0.996	0.998	0.998	0.996	0.998
CPR§	Quantitative	0.997	0.996	0.998	0.997	0.996	0.998
WBIC¶		151,185.41			150,049.69		

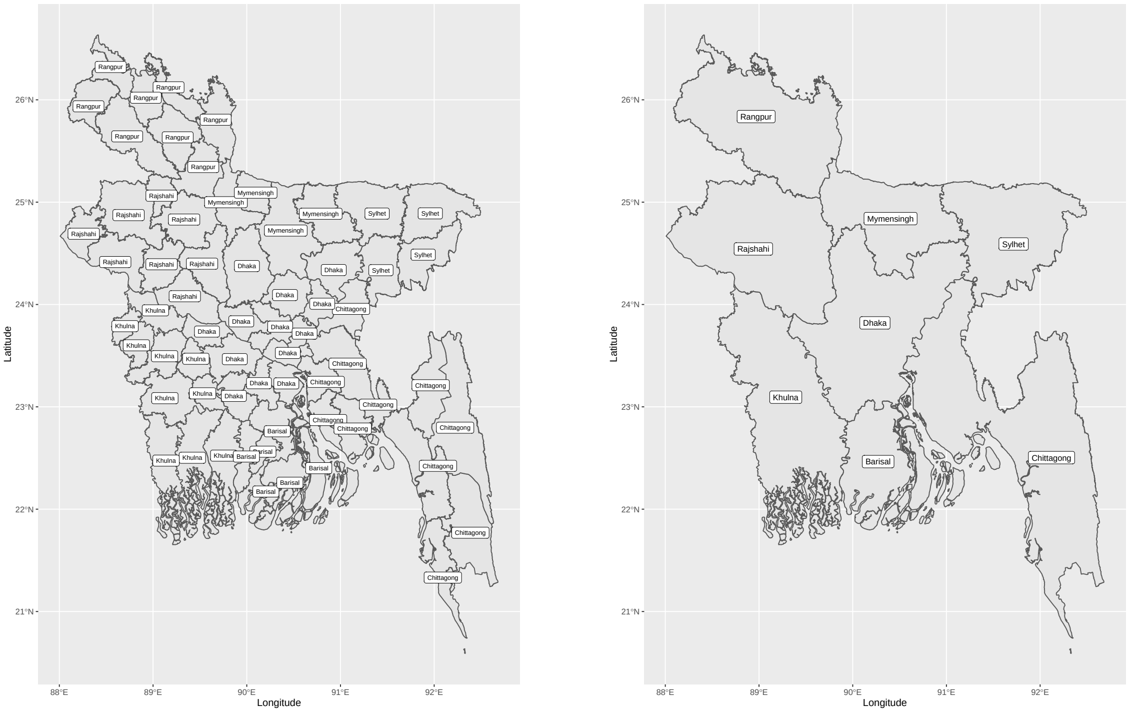

(a) Districts (b) Divisions

Figure F3. Districts and divisions of Bangladesh.

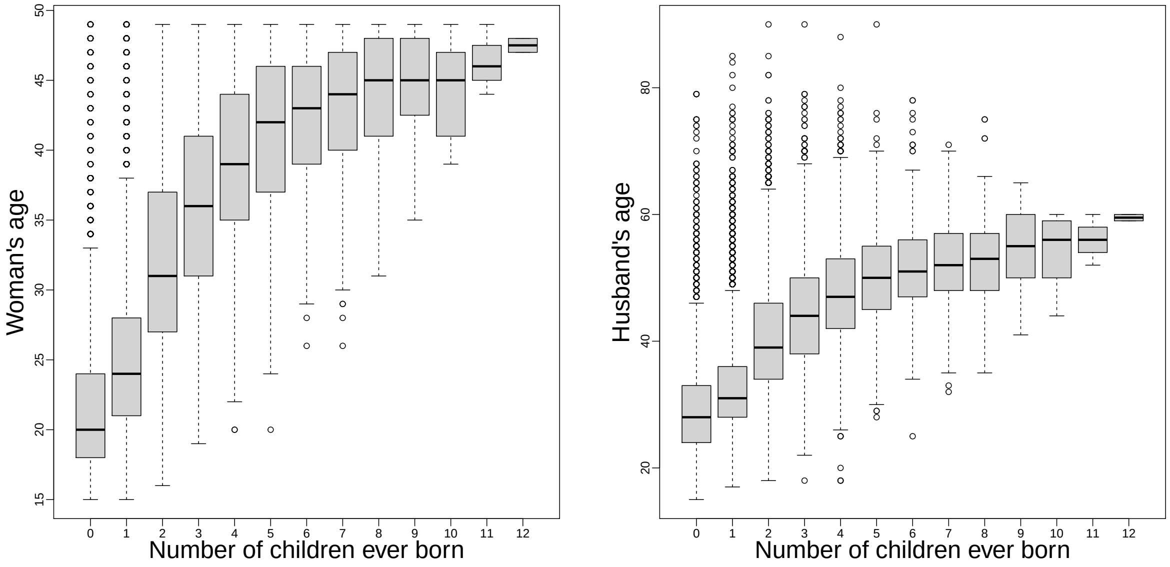

(a) Woman's age (b) Husban's age

Figure F4. Boxplots of woman's and husband's ages against the number of children ever born.
